# A81 TRANSITION PRACTICES FROM PEDIATRIC TO ADULT CARE OF CHILDREN LIVING WITH CROHN’S DISEASE IN QUEBEC

**DOI:** 10.1093/jcag/gwab049.080

**Published:** 2022-02-21

**Authors:** R Gong, R Kafyeke, B Bah, A Weber, M Morsa, C Deslandres, P Jantchou

**Affiliations:** 1 Universite de Montreal, Montreal, QC, Canada; 2 Centre Hospitalier de l’Universite de Montreal, Montreal, QC, Canada; 3 Universite Sorbonne Paris Nord, Villetaneuse, Île-de-France, France; 4 Service de gastro-entérologie, CHU Sainte-Justine, Montréal, QC, Canada; 5 Pediatrics, Sainte Justine University Hospital, Montreal, QC, Canada

## Abstract

**Background:**

Nearly 25% of Crohn’s disease cases are diagnosed during childhood. Among them, several adolescents may have extensive or complex disease implying specific needs during transition to adult care.

**Aims:**

The primary aim was to describe current transition practices from pediatric to adult care in patients diagnosed with Crohn’s disease at CHU Sainte-Justine. The secondary aim was to determine factors that influenced the type of adult health centers (academic vs non-academic) to which patients were referred.

**Methods:**

This single center study included patients diagnosed with Crohn’s disease at CHU Sainte-Justine between 2009 and 2019. Adult centers were separated into five categories: academic centers in Montreal (CHU-Mtl) and outside of Montreal (CHU), non-academic centers in Montreal (CHG-Mtl) and outside of Montreal (CHG), and other centers. The following factors influencing the transfer to an academic center were analyzed in a multivariate logistic regression model: age at diagnosis, gender, disease location, disease activity: relapses, hospitalizations, emergency room (ER) visits, and place of residence.

**Results:**

A total of 366 patients were included: 44% female, median (IQR) age at transfer 18.0 (17.9–18.4). Among them, 169 (48%) were transferred to CHU-Mtl, 144 (39%) to CHG, 22(6%) to CHU, 4 (1%) to CHG-Mtl, 27 (7%) to other centers. There was a significant increase in the annual number of patients referred to CHG and CHU-Mtl across the decade, compared to other centers. Patients transferred to CHU-Mtl had more relapses per year (mean (SD) 0.8 (0.5) versus patients transferred to CHU, CHG and CHG-Mtl, p=0.0348), and 57% (N=97) of patients sent to CHU-Mtl had already visited the ER, as compared to 54%, 40% and 25% for CHU, CHG and CHG-Mtl respectively (p=0.0258). However, gender, age at diagnosis, maintenance treatment, number and duration of hospitalisations, extraintestinal manifestations, perianal inflammation or extensive disease location did not correlate with the type of adult center. Place of residence played a role in the choice of adult center: 56% (N= 95) of patients transferred to CHU-Mtl lived in Montreal (p<0.0001).

**Conclusions:**

Clinical evolution and disease burden have an impact on the type of adult center. Efforts should be put to understand patient factors associated with the transfer to an academic vs non-academic center, for a better utilization of healthcare resources and adequate patient quality of life during transition.

**Funding Agencies:**

NonePrincipal researcher funds

